# Acute myocardial infarction is associated with endothelial glycocalyx and cell damage and a parallel increase in circulating catecholamines

**DOI:** 10.1186/cc12532

**Published:** 2013-02-22

**Authors:** Sisse R Ostrowski, Sune H Pedersen, Jan S Jensen, Rasmus Mogelvang, Pär I Johansson

**Affiliations:** 1 Section for Transfusion Medicine at Capital Region Blood Bank, Rigshospitalet, Blegdamsvej 9, Copenhagen, DK-2100, Denmark; 2 Department of Cardiology P, Gentofte Hospital, Niels Andersens Vej 65, Hellerup, DK-2900, Denmark; 3 Clinical Institute of Surgery and Internal Medicine, Faculty of Health Science at University of Copenhagen, Blegdamsvej 3B, Copenhagen, DK-2200, Denmark; 4 Department of Cardiology, Rigshospitalet, Blegdamsvej 9, Copenhagen, DK-2100, Denmark; 5 Department of Surgery, Center for Translational Injury Research (CeTIR) at University of Texas Medical School at Houston, 6410 Fannin Street, Houston, TX 77030, USA

## Abstract

**Introduction:**

Excessive sympathoadrenal activation in critical illness contributes directly to organ damage, and high concentrations of catecholamines damage the vascular endothelium. This study investigated associations between potential drivers of sympathoadrenal activation, circulating catecholamines and biomarkers of endothelial damage and outcome in ST segment elevation myocardial infarction (STEMI)-patients, hypothesizing that the catecholamine surge would reflect shock degree and correlate with biomarkers of endothelial damage.

**Methods:**

This was a prospective study of 678 consecutive STEMI-patients admitted to a single high-volume invasive heart centre for primary percutaneous coronary intervention (pPCI) from September 2006 to July 2008. Blood samples were drawn immediately before pPCI. Plasma adrenaline, noradrenaline, syndecan-1 and thrombomodulin were measured retrospectively with complete data in 571 patients (84%). Median follow-up time was 28 (IQR 23 to 34) months. Follow-up was 99.7% complete. Outcomes were all-cause and cardiovascular mortality, re-myocardial infarction and admission due to heart failure.

**Results:**

Circulating noradrenaline and adrenaline correlated weakly but independently with syndecan-1 (rho = 0.15 and rho = 0.13, both *P *<0.01) and thrombomodulin (rho = 0.11 and rho = 0.17, both *P *<0.01), biomarkers of glycocalyx and endothelial cell damage, respectively. Considering biomarkers, patients with shock pre-pPCI had higher adrenaline and syndecan-1 and patients admitted to ICU post-pPCI had higher syndecan-1 (all *P *<0.05), and in the patients with shock (*n *= 51) catecholamines correlated strongly with thrombomodulin and syndecan-1 (rho = 0.31 to 0.42, all *P *<0.05). During follow-up, 78 (14%) patients died (37 cardiovascular deaths) and 65 (11%) were admitted with heart failure. By multivariate Cox proportional hazards analyses, one quartile higher plasma adrenaline was weakly but independently associated with both 30-day and long term mortality and heart failure (30-day all-cause mortality hazard ratio (95% CI) 1.39 (1.01 to 1.92), *P *= 0.046; 30-day heart failure 1.65 (1.17 to 2.34), *P *= 0.005; and long-term cardiovascular mortality 1.49 (1.08 to 2.04), *P *= 0.014). Furthermore, one quartile higher syndecan-1 was also weakly but independently associated with long-term all cause mortality (1.26 (1.02 to 1.57), *P *= 0.034).

**Conclusions:**

In STEMI patients treated with pPCI, catecholamines correlated weakly with biomarkers of endothelial damage, with the strongest correlations and highest adrenaline and syndecan-1 levels in patients with shock. Furthermore, adrenaline and syndecan-1 were weakly but independently associated with mortality and heart failure. Acute myocardial infarction appears to cause significant endothelial cell and glycocalyx injury and a parallel increase in circulating catecholamines.

## Introduction

Excessive sympathoadrenal activation is a hallmark of acute critical illness and the accompanying increase in circulating catecholamines induces widespread dose-dependent effects on metabolism and the vascular system [1-3]. This 'fight-or-flight' response may, however, become maladaptive and contribute to organ damage [2-4], and in high concentrations, catecholamines directly damage the vascular endothelium resulting in local edema, endothelial cell swelling, necrosis and progressive de-endothelialization [5,6]. In two independent cohorts of trauma patients, we recently reported that plasma adrenaline correlated strongly with circulating biomarkers of endothelial activation and damage [7,8] and that the plasma adrenaline level was independently associated with circulating syndecan-1 [9], a recognized biomarker of endothelial glycocalyx degradation [10]. Furthermore, non-surviving trauma patients had increased levels of adrenaline and syndecan-1 and both predicted 30-day mortality [7,9].

Acute myocardial ischemia and infarction (MI) immediately activates the sympathoadrenal system resulting in excessive increases in circulating levels of adrenaline and noradrenaline [11,12], the latter through direct release from the infarcted myocardium [12]. Apparently, catecholamines induce opposite directed effects on the endothelium (progressive activation and damage) [4-6] and circulating blood (initial hypercoagulability followed by progressive hypocoagulability and hyperfibrinolysis) [4,13-18], and we infer that this reflects an evolutionary adapted response aimed at maintaining blood flow through a damaged and procoagulant microvasculature in the (shocked) critically ill patient [4]. In accordance with this notion, patients with cardiac arrest present with hyperfibrinolysis on-scene (extreme hypocoagulability as a result of excessive endothelial release of pro-fibrinolytic factors) [19] and increased circulating levels of endothelial derived biomarkers (syndecan-1, thrombomodulin, sICAM-1, sVCAM-1, sE-selectin) in the hours after cardiopulmonary resuscitation [20,21]. Although acute critical illness, with shock/hypotension, ischemia and reperfusion, massive tissue injury and systemic infection/inflammation, activates and potentially damages the endothelium [8,9,20-27], the relative contribution of the concurrent neurohumoral, including sympathoadrenal, activation to the endothelial injury and ensuing (multiple) organ failure [2-4] is not known.

MI patients have varying degrees of hypotension/shock and increases in circulating catecholamines, in the absence of massive tissue injury. Given this, the aim of the present study was to investigate associations I) between potential drivers of sympathoadrenal activation and/or endothelial damage and II) between circulating levels of catecholamines and biomarkers of endothelial glycocalyx (syndecan-1 [10]) and cell (soluble thrombomodulin, sTM [28-30]) damage and organ failure/outcome, in patients with ST elevation MI (STEMI). We hypothesized that shock and high catecholamine levels would be associated with evidence of enhanced damage to the glycocalyx and endothelium and that high levels of both catecholamines and endothelial derived biomarkers would be associated with a poor outcome.

Here, we report that circulating adrenaline and noradrenaline levels in STEMI patients treated with percutaneous primary coronary intervention (pPCI) correlated weakly with syndecan-1 and sTM, biomarkers of glycocalyx and endothelial cell damage, respectively, with the strongest correlations and highest levels of adrenaline and syndecan-1 in patients with shock prior to pPCI. Furthermore, adrenaline was independently associated with short-and long-term mortality and heart failure (HF), and syndecan-1 was independently associated with long-term mortality. These findings indicate that acute MI causes significant endothelial glycocalyx and cell injury and a parallel increase in circulating catecholamines and they support the notion that a dose-dependent association exists between disease severity, sympathoadrenal activation and endothelial damage.

## Materials and methods

### Study population

A total of 730 patients were treated with pPCI for STEMI at Gentofte University Hospital from September 2006 to September 2008 [31]. Plasma was retrospectively analyzed for adrenaline, noradrenaline, syndecan-1 and sTM in 678, 677, 628 and 574 patients, respectively, with complete measurements of all 4 biomarkers in 571 patients, that is, the present study cohort. If patients had more than one pPCI-procedure within this period, the first procedure was defined as the index-procedure.

Inclusion criteria and STEMI definition are as follows: chest pain >30 minutes and <12 hours and cumulative persistent ST-segment elevation ≥4 mm in at least two contiguous precordial ECG-leads or ≥2 mm in at least two contiguous limb ECG-leads. A significant increase in troponin I (TnI, >0.5 μg/l) was required for inclusion in the present study. The study was approved by the local scientific ethical committee and The Danish Data Protection Agency, and complied with the Second Declaration of Helsinki. Written informed consent was obtained from all patients.

### Baseline-and procedural data

Prospectively collected data (baseline, disease severity) are as follows: hypertension, hypercholesterolemia and diabetes (patients taking blood-pressure-, cholesterol-or glucose-lowering drugs, respectively, on admission and for the latter, with fasting plasma glucose concentration ≥7 mmol/L or non-fasting plasma glucose concentration ≥11.1 mmol/L); previous diagnosis of MI; multivessel disease (two-or three vessel-disease); complex lesions (type C-lesions); stenosis degree (1 or >1 infarcted segments); number of lesions (1 to 5); presence of shock prior to pPCI (systolic blood pressure ≤90 mmHg, need for vasopressor therapy and/or cardiopulmonary resuscitation pre-hospital or at admission) and admission to the ICU prior to hospital discharge.

TnI-levels were measured at admission and six hours and twelve hours after admission; the peak TnI level was used in the statistical analyses. C-reactive protein (CRP), estimated glomerular filtration tate (eGFR) and standard hematology analyses (hemoglobin, platelet, leukocyte and neutrophil counts) were measured at admission.

The pPCI procedure was performed according to contemporary interventional guidelines using pre-treatment with unfractionated heparin, acetyl salicylic acid and clopidogrel. Subsequent medical treatment included anti-ischemic, lipid-lowering and anti-thrombotic drugs according to current treatment guidelines.

### Follow-up and study end points

Follow-up was 99.7% complete (two patients were lost to follow-up due to emigration). The study endpoints were all-cause mortality, cardiovascular (CV) mortality, re-MI and admission with clinical signs of HF (dyspnea, fatigue, edema/stasis) combined with a discharge-diagnosis of HF. Follow-up data on mortality were collected from the National Person Identification Registry which holds information on vital status. Follow-up data on re-MI and admission with HF were collected using hospital source data as well as data from the Danish National Board of Health's National Patient Registry, using International Classifications of Diseases, tenth revision (ICD-10) codes. Median follow-up time was 28 months (IQR 23 to 34).

### Blood sampling

Peripheral arterial blood was drawn from the femoral sheath at the beginning of the procedure. Blood was allocated to different containers including 4 ml ethylenediaminetetraacetic acid (EDTA) tubes and was centrifuged at 10,000 RPM for 10 minutes within 30 minutes of collection. Plasma was stored in NuncCryo tubes (Nunc, Roskilde, Denmark) at-80°C.

### Enzyme linked immunosorbent assay (ELISA) measurements

Biomarkers of sympathoadrenal activation (adrenaline, noradrenaline) and endothelial glycocalyx (syndecan-1) and endothelial cell damage (sTM) were measured by commercially available immunoassays in EDTA plasma according to the manufactures' recommendations: for adrenaline and noradrenaline (2-CAT ELISA^FAST TRACK^, Labor Diagnostica Nord GmbH & Co. KG, Nordhorn, Germany; lower limit of detection (LLD) 10 pg/ml (adrenaline) and 50 pg/ml (noradrenaline), respectively; for syndecan-1 (Diaclone SAS, Besancon, France; LLD 4.94 ng/ml); and for sTM (Nordic Biosite, Copenhagen, Denmark; LLD 0.38 ng/ml). Values below LLD were recorded as the LLD value (*n *= 62, 78, 8 and 4 for adrenaline, noradrenaline, syndecan-1 and sTM, respectively).

### Statistics

Statistical analysis was performed using SAS 9.1 (SAS Institute Inc., Cary, NC, US). Data from patients stratified according to adrenaline or syndecan-1 quartiles were compared by Kruskal-Wallis and Chi-square/Fisher´s exact tests, as appropriate, and by Bonferroni corrected Wilcoxon Rank Sum and Chi-square/Fischer exact post-hoc tests. Biomarker levels in patients stratified according to shock prior to pPCI or ICU admission before discharge were compared by Wilcoxon Rank Sum tests. Correlations between biomarkers were investigated by Spearman´s correlations. The contribution of baseline variables to the variation in syndecan-1 and sTM levels was investigated by univariate and multivariate linear regression analyses. The predictive value of quartiles of adrenaline, noradrenaline, syndecan-1 and sTM for 30-day and long-term all-cause mortality, CV mortality, re-MI and HF were investigated by univariate and multivariate Cox proportional hazards analyses. To maintain robust models, only one variable per five events was allowed in the multivariable Cox analyses of each endpoint. Variables with the lowest *P*-value in univariate Cox analysis were included until the maximum allowed number of variables was reached. Data are presented as medians with inter IQRs. *P*-values <0.05 were considered significant.

## Results

### Study patients

Baseline characteristics and outcome in all patients and in patients stratified according to adrenaline quartiles are presented in Table 1. With increasing adrenaline quartile, the noradrenaline level increased progressively and more patients presented with shock prior to pPCI, developed HF or died during follow-up. Patients in the highest adrenaline quartiles tended to have lower eGFR and more complex lesions (Type C).

**Table 1:** Demography, baseline data and outcome in 571 consecutive STEMI patients treated with primary PCI

		All patients	Adrenaline Q1	Adrenaline Q2	Adrenaline Q3	Adrenaline Q4	*P*-value
		**Number = 571**	**Number = 143**	**Number = 143**	**Number = 142**	**Number = 143**	

**Demography and cardiovascular risk factors**							
Age	years	63 (55-72)	64 (54-74)	64 (55-73)	62 (56-70)	64 (57-71)	NS
Male gender	n (%)	419 (73%)	103 (72%)	97 (68%)	112 (79%)	107 (75%)	0.192
Hypertension	n (%)	197 (35%)	51 (36%)	49 (34%)	51 (36%)	46 (32%)	NS
Diabetes	n (%)	55 (10%)	17 (12%)	13 (9%)	14 (10%)	11 (8%)	NS
Current smoker	n (%)	285 (50%)	70 (49%)	77 (54%)	70 (49%)	68 (48%)	NS
Previous MI	n (%)	33 (6%)	10 (7%)	8 (6%)	6 (6%)	4 (4%)	NS
BMI	kg/m^2^	26 (24-29)	26 (23-30)	26 (23-28)	26 (24-30)	26 (24-29)	NS
**Pre-and in-hospital symptoms**							
Symptom-to-balloon time	min	200 (134-330)	195 (137-325)	225 (133-375)	205 (131-360)	180 (120-299)	0.172
Door-to-balloon time	min	60 (31-110)	50 (30-101)	70 (32-120)	63 (36-110)	60 (31-103)	NS
**Clinical presentation**							
Shock prior to pPCI	n (%)	51 (11%)	7 (6%)	9 (8%)	13 (12%)	22 (18%)	**0.016^a^**
Systolic BP	mmHg	135 (115-150)	131 (115-150)	135 (116-150)	138 (119-150)	130 (110-150)	NS
Diastolic BP	mmHg	80 (70-90)	80 (70-90)	80 (70-90)	80 (70-90)	80 (70-90)	NS
Left ventricular ejection fraction	%	40 (30-50)	40 (30-50)	40 (30-50)	40 (30-50)	35 (25-45)	NS
eGFR at admission	ml/min	73 (60-88)	76 (64-89)	74 (56-88)	74 (62-88)	70 (54-85)	0.052
**Hematology and biomarkers of myocardial necrosis and inflammation**							
Hemoglobin	mmol/L	8.7 (8.1-9.3)	8.7 (8.1-9.2)	8.8 (8.2-9.3)	8.7 (8.1-9.3)	8.7 (8.2-9.2)	NS
Platelet count	x 10^9^/L	282 (234-333)	280 (242-346)	292 (241-345)	284 (233-317)	271 (227-332)	NS
Leukocyte count	x 10^9^/L	12.2 (9.8-15.1)	11.6 (9.3-14.5)	12.3 (10.1-15.3)	12.5 (10.4-14.9)	12.4 (9.9-15.5)	NS
Neutrophil count	x 10^9^/L	9.2 (6.8-11.8)	8.8 (6.7-11.3)	8.9 (7.2-11.6)	9.5 (7.2-12.2)	9.3 (6.3-12.2)	NS
Peak Troponin I	µg/L	90 (28-244)	98 (24-242)	116 (33-261)	60 (21-213)	95 (38-255)	NS
CRP	mg/L	3 (1-9)	4 (1-11)	3 (2-9)	3 (1-7)	3 (2-10)	NS
**Sympathoadrenal activation and endothelial damage**							
Adrenaline	pg/ml	59 (25-145)	13 (10-20)	37 (29-50)	83 (73-94)	283 (213-501)	**<0.0001**^abcdef^
Noradrenaline	pg/ml	191 (90-454)	117 (57-236)	150 (78-294)	214 (108-498)	441 (229-1,180)	**<0.0001**^abdef^
Syndecan-1	ng/ml	92 (52-165)	84 (46-156)	81 (47-162)	95 (55-168)	101 (52-169)	NS
Soluble thrombomodulin	ng/ml	2.2 (1.6-3.1)	2.1 (1.6-3.1)	2.3 (1.7-3.2)	2 (1.5-2.8)	2.2 (1.7-3.2)	**0.040**
**Infarction type**							
Lesion type	A	83 (15%)	18 (13%)	23 (16%)	27 (19%)	15 (10%)	0.194
	B	209 (37%)	52 (36%)	56 (39%)	54 (38%)	47 (33%)	
	C	278 (49%)	73 (51%)	63 (44%)	61 (43%)	81 (57%)	
Infarct related artery	LAD	262 (46%)	62 (43%)	66 (46%)	60 (42%)	74 (52%)	0.092
	RCA	246 (43%)	62 (43%)	54 (38%)	69 (49%)	61 (43%)	
	Cx	58 (10%)	19 (13%)	20 (14%)	13 (9%)	6 (4%)	
	LM	3 1%)	0 (0%)	1 (1%)	0 (0%)	2 (1%)	
	Graft	1 (0%)	0 (0%)	0 (0%)	0 (0%)	1 (1%)	
Complex lesion	n (%)	278 (49%)	73 (51%)	63 (44%)	61 (43%)	81 (57%)	0.071
Multivessel disease	n (%)	160 (28%)	47 (33%)	35 (24%)	37 (26%)	41 (29%)	NS
**Clinical outcome**							
ICU admission after pPCI	n (%)	30 (6%)	8 (7%)	5 (4%)	8 (7%)	9 (7%)	NS
Follow-up time	months	28 (23-34)	-	-	-	-	
All-cause mortality	n (%)	78 (14%)	14 (10%)	23 (16%)	10 (7%)	31 (22%)	**0.001^af^**
CV mortality	n (%)	37 (7%)	3 (2%)	10 (7%)	6 (4%)	18 (13%)	**0.002^a^**
Re-MI	n (%)	46 (8%)	14 (10%)	10 (7%)	11 (8%)	11 (8%)	NS
Admission due to heart failure	n (%)	65 (11%)	9 (6%)	21 (15%)	12 (8%)	23 (16%)	**0.023**

Data from all patients and patients stratified according to adrenaline quartiles are displayed. Data are presented as medians (IQR) or number (%), with *P*-values shown for variables with *P *<0.2, and in bold for *P *<0.05. Adrenaline quartiles (Q1 to Q4) were compared by Kruskal-Wallis and Chi-square/Fischer exact tests, as appropriate, and by Bonferroni corrected Wilcoxon Rank Sum and Chi-square/Fisher exact post-hoc tests. Significant post-hoc test difference (*P *<0.05) between: ^a^Q1 and Q4, ^b^Q1 and Q3, ^c^Q1 and Q2, ^d^Q2 and Q4, ^e^Q2 and Q3, ^f^Q3 and Q4.

BMI, body mass index; BP, blood pressure; CRP, C-reactive protein; CV, cardiovascular; Cx, circumflex; eGFR, estimated glomerular filtration rate; LAD, left anterior descending; LM, left main; MI, myocardial infarction; n, number; NS, non-significant; PCI, primary coronary intervention; RCA, right coronary artery; STEMI, ST elevation myocardial infarction.

**Figure 1:**
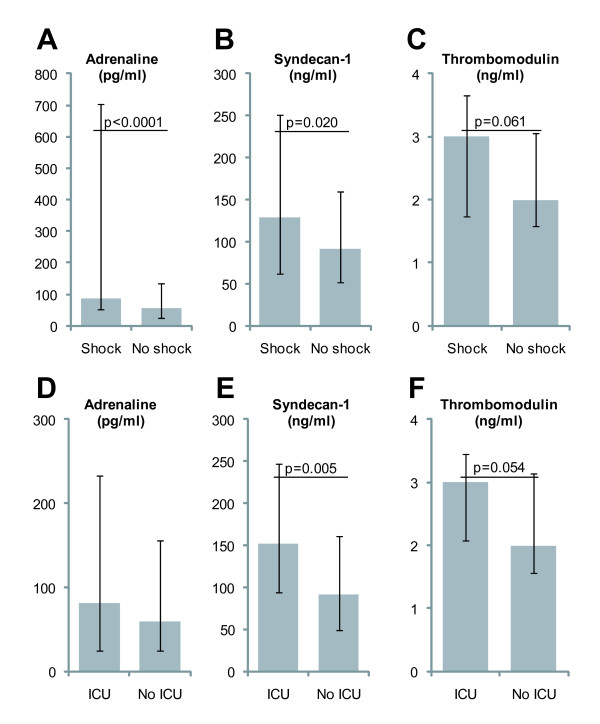
**Plasma levels of adrenaline, syndecan-1 and thrombomodulin in STEMI-patients with or without shock prior to primary PCI (*n *= 51 with shock, A-C) or ICU admission before discharge (*n *= 30 admitted to ICU, D-F)**. Medians with inter quartile ranges (IQR) are shown for adrenaline (pg/ml, A and D), syndecan-1 (ng/ml, B and E) and thrombomodulin (ng/ml C and F). *P*-values for Wilcoxon Rank Sum tests are shown. PCI, primary coronary intervention; STEMI, ST elevation myocardial infarction.

When comparing biomarker levels in patients stratified according to shock prior to pPCI or ICU admission before discharge, adrenaline levels were higher in patients with shock (Figure 1A) and syndecan-1 levels were higher in patients with shock or ICU admission before discharge (Figure 1B,E). sTM tended to be higher in patients with shock or ICU admission (Figure 1C,F). eGFR was lower in patients with shock (*P *<0.001) or ICU admission (*P *<0.001) whereas noradrenaline, CRP and peak-TnI levels were comparable in patients with shock or ICU admission (data not shown).

Given that syndecan-1 was increased in both shocked and ICU patients, patients stratified according to syndecan-1 quartiles were investigated. With increasing syndecan-1 quartile (33 ng/ml (IQR 19 to 40), 69 ng/ml (IQR 59 to 79), 123 ng/ml (IQR 104 to 144) and 248 ng/ml (IQR 206 to 299) in Q1 to Q4, respectively), more patients had shock prior to PCI (Q1 to 4: 10%, 5%, 9% and 19%, *P *= 0.011) and eGFR decreased (Q1 to 4: 74 ml/min, 76 ml/min, 72 ml/min and 71 ml/min, *P *= 0.023) whereas neutrophils (Q1 to 4: 8.6 × 10^9^/L, 9.4 × 10^9^/L, 9.4 × 10^9^/L and 9.6 × 10^9^/L, *P *= 0.038), platelet count (Q1 to 4: 271 × 10^9^/L, 283 × 10^9^/L, 272 × 10^9^/L and 296 × 10^9^/L, *P *= 0.036), CRP (Q1 to 4: 3 mg/ml, 3 mg/ml, 4 mg/ml and 4 mg/ml, *P *= 0.002) and sTM (Q1 to 4: 1.8 ng/ml, 2.1 ng/ml, 2.3 ng/ml and 2.8 ng/ml, *P *<0.0001) increased. With increasing syndecan-1 quartile more patients went to the ICU post-PCI (Q1 to 4: 3%, 3%, 8% and 11%, *P *= 0.027) and more patients died (30-day all-cause mortality Q1 to 4: 3%, 3%, 5% and 12%, *P *<0.002 and long-term all-cause mortality Q1 to 4: 9%, 10%, 12% and 23%, *P *<0.004) or were admitted with HF (30-day HF Q1 to 4: 2%, 5%, 6% and 10%, *P *= 0.008 and long-term HF Q1 to 4: 6%, 11%, 13% and 15%, *P *= 0.015) (data not shown).

### Correlations between catecholamines, endothelial damage and markers of shock and infarction degree

**Figure 2:**
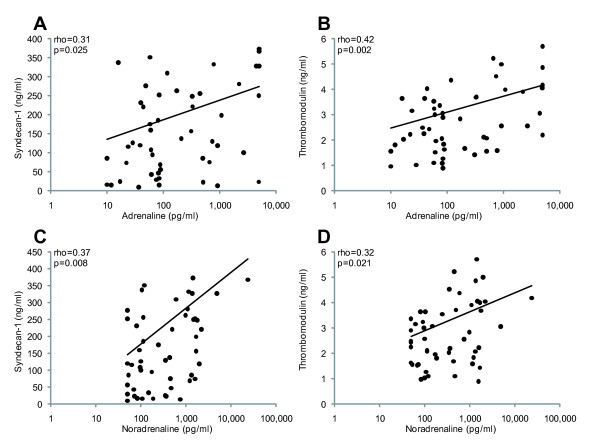
**Scatter plots showing correlations between plasma levels of adrenaline and noradrenaline and syndecan-1 and thrombomodulin in STEMI-patients with shock prior to primary PCI (*n *= 51)**. **A**) adrenaline versus syndecan-1, **B**) noradrenaline versus syndecan-1, **C**) adrenaline versus thrombomodulin and **D**) noradrenaline versus thrombomodulin. Rho and *P*-values are shown for Spearman correlations. PCI, primary coronary intervention; STEMI, ST elevation myocardial infarction.

Adrenaline and noradrenaline were highly positively correlated (rho = 0.43, *P *<0.001) and both adrenaline and noradrenaline correlated weakly positively with syndecan-1 (rho = 0.13, *P *= 0.003 and rho = 0.15, *P *<0.001, respectively) and sTM (rho = 0.17, *P *<0.001 and rho = 0.11, *P *= 0.006, respectively) when investigated in all patients. Since shock was associated with increased levels of adrenaline and syndecan-1, correlations between catecholamines and biomarkers of endothelial damage were investigated in patients stratified according to the presence or absence of shock prior to pPCI. In patients with shock, adrenaline and noradrenaline correlated even more strongly with syndecan-1 and sTM (Figure 2A-D) whereas they did not correlate in patients without shock (data not shown).

In all patients, systolic blood pressure correlated negatively with adrenaline (rho =-0.14, *P *<0.001) and noradrenaline (rho =-0.10, *P *= 0.020) but positively with eGFR (rho = 0.11, *P *= 0.012). eGFR correlated negatively with adrenaline (rho =-0.11, *P *= 0.006), noradrenaline (rho =-0.13, *P *= 0.002) and sTM (rho =-0.32, *P *<0.001). Peak TnI correlated positively with both noradrenaline (rho = 0.09, *P *= 0.026) and sTM (rho = 0.13, *P *= 0.003).

### Variables associated with biomarkers of endothelial damage

By univariate linear regression, higher adrenaline and noradrenaline, female gender, diabetes, shock prior to pPCI and lower eGFR were associated with higher syndecan-1 levels but in the multivariate model only noradrenaline, diabetes and shock prior to pPCI were independently associated with syndecan-1 (Table 2). Variables associated univariately with higher sTM were higher adrenaline, noradrenaline, age and peak TnI, female gender, non-smoking status, diabetes, multivessel disease and lower BMI and eGFR whereas only adrenaline, diabetes, BMI and eGFR were independently associated with sTM (Table 3). Although syndecan-1 and sTM levels were strongly intercorrelated (rho = 0.28, *P *<0.0001), they were not included in the multivariate analyses since they both, by different means, reflect endothelial damage.

**Table 2:** Variables associated with plasma syndecan-1 by univariate and multivariate (backwards selection) linear regression analysis **in 571 **consecutive STEMI patients treated with primary PCI

		Univariate			Multivariate		
					**R^2^ = 0.05**		

		**β (95%CI)**	**t**	***P***	**β (95%CI)**	**t**	***P***

Adrenaline	100 pg/ml	1.64 (0.58 to 2.71)	3	**0.003**			NS
Noradrenaline	100 pg/ml	1.20 (0.57 to 1.84)	4	**<0.001**	1.12 (0.46 to 1.78)	3	**0.001**
Male gender	yes	-24 (-41 to-8)	-3	**0.004**			NS
Diabetes	yes	29 (4 to 54)	2	**0.024**	31 (3 to 58)	2	**0.032**
eGFR	ml/min	-0.41 (-0.72 to-0.10)	-3	**0.009**			NS
Peak TnI	µg/L	0.04 (0 to 0.08)	2	0.051			NS
Shock prior to pPCI	yes	43 (17 to 69)	3	**0.002**	35 (8 to 61)	3	**0.010**

Regression coefficients (β) with 95% confidence intervals (95%CI), t-and *P*-values and R^2 ^displayed for the multivariate models. *P*-values are shown in bold for variables with *P *<0.05. Predicted change in syndecan-1 (pg/ml) associated with one unit increase in adrenaline, noradrenaline, eGFR and peak TnI, being male or having diabetes or shock prior to pPCI. eGFR, estimated glomerular filtration rate; pPCI, percutaneous primary coronary intervention; STEMI, ST elevation myocardial infarction; TnI, troponin I.

**Table 3:** Variables associated with plasma thrombomodulin by univariate and multivariate (backwards selection) linear regression analysis in 571 consecutive STEMI patients treated with primary PCI

		Univariate			Multivariate		
					**R^2 ^= 0.19**		

		**β (95%CI)**	**t**	***P***	**β (95%CI)**	**t**	***P***

Adrenaline	100 pg/ml	0.03 (0.02 to 0.05)	4	**<0.0001**	0.02 (0.01 to 0.03)	3	**0.003**
Noradrenaline	100 pg/ml	0.01 (0.00 to 0.02)	3	**0.006**			NS
Age	years	0.02 (0.02 to 0.03)	6	**<0.0001**			NS
Male gender	yes	-0.37 (-0.60 to-0.15)	-3	**0.001**			NS
BMI	kg/m^2^	-0.03 (-0.06 to-0.01)	-3	**0.002**	-0.04 (-0.06 to-0.02)	-4	**0.001**
Current smoker	yes	-0.41 (-0.61 to-0.21)	-4	**<0.0001**			NS
Diabetes	yes	0.38 (0.04 to 0.72)	2	**0.029**	0.45 (0.13 to 0.76)	3	**0.006**
eGFR	ml/min	-0.02 (-0.02 to-0.02)	-10	**<0.0001**	-0.02 (-0.02 to-0.01)	-10	**<0.0001**
Peak TnI	µg/L	0.00 (0.00 to 0.00)	3	**0.008**			NS
Multivessel disease	yes	0.32 (0.10 to 0.55)	3	**0.004**			NS

Regression coefficients (β) with 95% confidence intervals (95%CI), t-and *P*-values and R^2 ^displayed for the multivariate models. *P*-values are shown in bold for variables with *P *<0.05. Predicted change in thrombomodulin (pg/ml) associated with one unit increase in adrenaline, noradrenaline, age, BMI, eGFR and peak TnI, being male, smoker or having diabetes or multivessel disease. BMI, body mass index; eGFR, estimated glomerular filtration rate; PCI, primary coronary intervention; STEMI, ST elevation myocardial infarction; TnI, troponin I.

### Catecholamines, endothelial damage and outcome

During a median follow-up of 28 months (IQR 23 to 34), 78 (14%) patients died (37 (7%) from CV causes), 46 (8%) had a re-MI and 65 (11%) were admitted with HF (Table 1). With regard to the time-point of deaths, 42% (*n *= 33) of all-cause fatal events occurred after 30 days.

**Figure 3:**
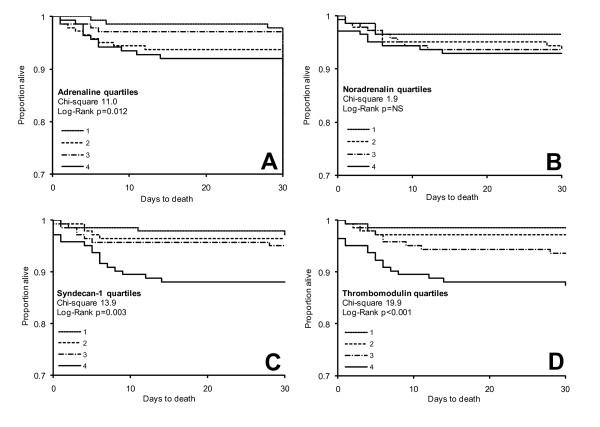
**Kaplan-Meier plots showing 30-day all-cause mortality in ST segment elevation myocardial infarction (STEMI) patients stratified into quartiles of circulating adrenaline, noradrenaline, syndecan-1 and sTM levels**. Survival times for the quartiles of each biomarker are shown for: adrenaline (**A**), noradrenaline (**B**), syndecan-1 (**C**) and soluble thrombomodulin (sTM, **D**). Chi-square and *P*-values for log-rank tests are shown.

Log-rank tests based on Kaplan-Meier curves for quartiles of adrenaline, syndecan-1 and sTM showed significant associations between high levels of each biomarker and increased 30-day and long-term all-cause and CV mortality (see Figure 3A-D for Kaplan-Meier plots of 30-day all-cause mortality). Kaplan-Meier curves for quartiles of adrenaline and noradrenaline also showed an association with 30-day and long-term admission for HF whereas syndecan-1 was only associated with 30-day HF and sTM only with long-term HF (data not shown).

When the associations between biomarkers and mortality were investigated by univariate Cox analyses, each increase in adrenaline, syndecan-1 or sTM quartiles was associated with increased risk of 30-day (Table 4) and long-term (all *P *<0.05, data not shown) all-cause and CV mortality and HF. After adjusting for conventional risk factors, adrenaline remained an independent predictor of 30-day all-cause mortality and HF (Table 4) and of long-term CV mortality (*P *= 0.014) and syndecan-1 remained an independent predictor of long-term all-cause mortality (*P *= 0.034). Noradrenaline was a univariate predictor of long-term CV mortality (*P *= 0.031) and long-term HF (*P *= 0.039). None of the investigated biomarkers could predict re-MI (Table 4 and data not shown).

**Table 4:** Cox Proportional Hazards models predicting 30-day all-cause and cardiovascular (CV) mortality and heart failure in 571 consecutive STEMI patients treated with primary PCI.

			Adrenaline		Noradrenaline		Syndecan-1		Thrombomodulin	
			**HR (95%CI)**	***P***	**HR (95%CI)**	***P***	**HR (95%CI)**	***P***	**HR (95%CI)**	***P***

All-cause mortality	30-day	uni	1.42 (1.03-1.96)	**0.032**		NS	1.74 (1.23-2.45)	**0.002**	2.13 (1.46-3.11)	**<0.0001**
		multi^a^	1.39 (1.01-1.92)	**0.046**		NS	1.29 (0.90-1.85)	0.166		NS

CV mortality	30-day	uni	1.45 (1.02-2.05)	**0.038**	1.27 (0.91-1.78)	0.167	1.59 (1.11-2.28)	**0.012**	2.39 (1.54-3.68)	**<0.0001**
		multi^b^	1.39 (0.98-1.96)	0.066		NS		NS	1.49 (0.93-2.38)	0.098

Re-MI	30-day	uni		NS		NS	0.71 (0.43-1.17)	0.176		NS
		multi^c^		NS		NS		NS		NS

Heart failure	30-day	uni	1.40 (1.02-1.92)	**0.041**	1.31 (0.96-1.80)	0.091	1.66 (1.19-2.32)	**0.003**	1.53 (1.10-2.12)	**0.011**
		multi^d^	1.65 (1.17-2.34)	**0.005**	1.29 (0.93-1.79)	0.135	1.38 (0.98-1.94)	0.069		NS

Hazards ratios (HR) with 95% confidence intervals (HR (95% CI)) and *P*-values associated with increased quartiles of adrenaline, noradrenaline, syndecan-1 or thrombomodulin are shown for univariate and multivariate analyses, with *P*-values in bold for variables with p <0.05. The applied multivariate Cox proportional hazards models (MV) included variables significant for 30-day events in the univariate analyses: ^a^(30-day all-cause mortality, *n *= 33) age (<0.001), systolic blood pressure (<0.0001), eGFR (<0.0001), peak TnI (<0.001), multivessel disease (<0.01); ^b^(30-day CV mortality, *n *= 28) systolic blood pressure (<0.001), eGFR (<0.0001), peak TnI (<0.0001), multivessel disease (<0.01); ^c^(30-day re-MI, *n *= 14) leukocyte count (<0.0001); ^d^(30-day heart failure, *n *= 33) age (<0.0001), BMI (<0.01), CV, cardiovascular; eGFR (<0.0001), CRP (<0.0001), peak TnI (<0.0001). BMI, body mass index; CRP, C-reaction protein; eGFR, estimated glomerular filtration rate; PCI, primary coronary intervention; Re-MI, Re myocardial infarction; STEMI, ST elevation myocardial infarction, TN1, troponin I.

Compared to the independent predictive value of conventional risk factors for outcome in MI patients in the present study (age, systolic BP, eGFR, peak TnI, CRP, multivessel disease, *P*-values displayed in Table 4 footer), the predictive value of the investigated biomarkers was weak.

## Discussion

Here, we report that circulating adrenaline and noradrenaline levels in STEMI-patients treated with pPCI correlated with syndecan-1 and sTM, biomarkers of glycocalyx and endothelial cell damage, respectively, with the strongest correlations, and highest adrenaline and syndecan-1 levels, in patients with shock. Furthermore, circulating levels of adrenaline and syndecan-1 were associated independently with short-and long-term mortality and HF and with long-term mortality, respectively. These findings demonstrate that acute MI appears to cause significant endothelial cell and glycocalyx injury and a parallel increase in circulating catecholamines. They also support the notion that a dose-dependent association between disease severity, sympathoadrenal activation and endothelial damage exists in critically ill non-trauma patients in accordance with that previously observed in trauma patients [7,9].

The vascular endothelium comprises a single layer of cells that lines every blood vessel in the body, covers a total surface area of 4 to 7,000 m^2^, totaling a weight of 1 kilogram [32]. On top of the endothelium lies the glycocalyx, an approximately 1 μm thick carbohydrate-rich structure with antiadhesive and anticoagulant properties that protects the endothelium and maintains vascular barrier function [22,23]. The endothelium is critically involved in the pathology of multiple diseases, in which there exist a well established association between endothelial dysfunction and/or damage and poor outcome [8,9,20-24]. In the present study, we investigated associations between potential drivers of sympathoadrenal activation, endothelial damage and outcome in patients with increased sympathoadrenal activation in the absence of massive tissue injury. Our findings confirmed, in a different cohort of patients, the previous finding in trauma patients of associations between shock, catecholamines, biomarkers of glycocalyx and endothelial cell damage and outcome [7-9]. Although the median circulating levels of adrenaline and noradrenaline in trauma patients (290 pg/ml (IQR 190 to 720) and 750 pg/ml (IQR 450 to 1,380), respectively) [7,9] are four-to five-fold higher than the median levels observed in STEMI-patients in this study (Table 1), STEMI-patients in the highest adrenaline quartile, representing the most critically ill and shocked patients, had catecholamine levels comparable to those observed in trauma patients [7,9]. Although we found correlations between catecholamines and biomarkers of endothelial damage in the whole patient cohort, these were weak and strongest in patients with shock, in accordance with the notion that a dose-response relationship may exist between catecholamine levels and infliction of endothelial injury [4-6].

Glycocalyx damage is associated with pathophysiologic sequels, such as capillary leakage and edema formation, accelerated inflammation, platelet activation and hypercoagulability and loss of vascular responsiveness [22]. It is becoming increasingly evident that glycocalyx damage represents the earliest stage of endothelial perturbation [22,23]. In STEMI-patients, several factors may contribute to glycocalyx damage, that is, ischemia-reperfusion injury, shock, inflammation (TNF-α), hyperglycemia, atrial natriuretic peptide and oxidized low density lipoprotein (LDL). Thus, in accordance with previous studies reporting that shock [21,25-27] and hyperglycemia/diabetes [7,23,33] are associated with glycocalyx damage, shock and diabetes were independently associated with circulating syndecan-1 in the present study (Table 2). With increasing syndecan-1 quartile, the neutrophil and platelet count and CRP increased whereas eGFR decreased. The finding that patients with shock displayed the strongest correlations between catecholamines and endothelial damage supports the notion that a threshold level exists above which catecholamines exert deleterious effects on the endothelial glycocalyx and cells.

We also found that syndecan-1 was associated independently with mortality and, to the best of our knowledge, this association has not been reported previously. Given the emerging evidence for devastating effects of glycocalyx damage in acute [9,22,27] and chronic [23] illness, including cardiovascular disease [21-23], this finding is notable.

In accordance with previous studies, higher age [34], male gender [34,35], non-smoking [34,35], diabetes [34] and impaired kidney function [36] were associated with higher circulating sTM, in addition to higher adrenaline levels, lower BMI, myocardial cell damage (TnI) and multivessel disease. Circulating adrenaline, BMI, diabetes and eGFR were independently associated with sTM (Table 3), indicating that both life style factors and acute critical illness may contribute to endothelial damage.

In the present study, more patients in the highest adrenaline quartile died or developed HF. Adrenaline was independently associated with mortality and HF which is in accordance with previous findings reporting (varying degrees of) association between early increases in circulating adrenaline levels and mortality in MI-patients [11,37,38]. From a pathophysiologic point of view, MI-induced sympathoadrenal activation may later aggravate chronic atherosclerosis [39]. In some studies the early adrenaline surge in MI-patients has been correlated with the extent of myocardial damage/infarct size [11,40] and left ventricular ejection fraction (LVFE) [38]. Although we could not replicate these associations, we found a borderline significant association between adrenaline levels and complex lesions (Table 1). It should be emphasized that the above findings do not prove any cause-effect relationship between early adrenaline levels, endothelial damage and poor outcome post-MI since it is expected that the most critically ill patients have the highest sympathoadrenal response and the poorest outcome. Also, it is possible that differences in the sympathoadrenal response attributed to gene polymorphisms in adrenergic receptors may in part explain our findings [41]. However, it is notable that the most critically ill and shocked MI patients presented with evidence of enhanced endothelial damage that correlated more strongly with catecholamines than in less sick patients. Whatever drivers among shock, ischemia-reperfusion injury, catecholamines, hyperglycemia, and so on that inflict the greatest endothelial damage, the magnitude of increase in endothelial derived biomarkers may be interpreted as a surrogate for concurrent organ damage and this may, in part, explain the negative predictive value associated with these biomarkers.

With regard to noradrenaline, this was a univariate predictor of long-term HF and CV mortality in this study. Although noradrenaline is a strong predictor of poor outcome in patients with asymptomatic left ventricular dysfunction [42] and chronic HF [3], the weak predictive value for outcome compared with adrenaline may both reflect that we investigated an early noradrenaline response, which may peak after PCI [43], and that noradrenaline release is much more heterogeneous compared to adrenaline release; for example, noradrenaline is released directly from the infarcted myocardium [12].

The results presented here are subject to the limitations inherent to observational studies and, therefore, do not allow independent evaluation of the cause and effect relationship between catecholamine levels, endothelial damage and outcome, so evidence of potential cause and effect relationships should come from adequately designed prospective trial(s). We found a relatively low prevalence of diabetes, previous MI and known HF prior to STEMI. Thus, care should be taken if the results are to be extrapolated to populations with very different distributions of potential risk factors and logistic facilities. Also, our geographical and organizational conditions may not necessarily apply to other countries and regions, and our findings should not be extrapolated to settings without high volume PCI centers. Finally, we did not have data on predictive ICU scores (Sequential Organ Failure Assessment, Acute Physiology and Chronic Health Evaluation, and so on) and we did not have data on previous β-adrenergic receptor blockers before admission.

## Conclusions

The present study found an association between shock, circulating catecholamine levels, biomarkers indicative of endothelial damage, and outcome in STEMI-patients treated with pPCI, in accordance with previous findings in trauma patients. Patients in shock presented with the highest levels of adrenaline and syndecan-1, and the correlations between catecholamines and endothelial-derived biomarkers were particularly strong in patients in shock. These findings demonstrate that acute MI appears to cause significant endothelial cell and glycocalyx injury and a parallel increase in circulating catecholamines. The predictive value of the endothelial-derived biomarkers for outcome in STEMI patients may indicate that these, in part, reflect the extent of acute (ischemic/catecholamine induced) organ damage, thereby providing a prognostic value.

## Key messages

• Sympathoadrenal activation is a hallmark of acute critical illness but this fight-or-flight response may become maladaptive and contribute to organ damage; in high concentrations catecholamines directly damage the vascular endothelium.

• In STEMI-patients circulating levels of adrenaline and noradrenaline correlated with levels of thrombomodulin and syndecan-1, biomarkers of endothelial cell and glycocalyx damage, with the strongest correlations in patients in shock.

• STEMI-patients with shock prior to PCI had the highest circulating adrenaline and syndecan-1 levels and patients admitted to ICU after PCI had the highest syndecan-1 levels.

• Circulating levels of adrenaline and syndecan-1 were associated independently with mortality and heart failure.

• Acute MI appears to cause significant endothelial cell and glycocalyx injury and a parallel increase in circulating catecholamines; these findings support the existence of a dose-dependent association between disease severity, sympathoadrenal activation and endothelial damage in critically ill non-trauma patients in accordance with that previously observed in trauma patients.

## Abbreviations

CV: cardiovascular; CRP: C-reactive protein; EDTA: ethylenediaminetetraacetic acid; eGFR: estimated glomerular filtration rate; ELISA: enzyme-linked immunosorbent assay; HF: heart failure; LDL: low density lipoprotein; LLD: lower limit of detection; MI: myocardial infarction; pPCI: primary percutaneous coronary intervention; sICAM-1: soluble intercellular adhesion molecule-1; sTM: soluble thrombomodulin; sVCAM-1: soluble vascular cell adhesion molecule-1; STEMI: ST elevation MI; TnI: troponin I.

## Competing interests

The authors declare that they have no competing interests.

## Authors' contributions

SRO participated in the conception of the study and in data acquisition, performed the statistical analysis and data interpretation and drafted the manuscript. SHP, JSJ and RM participated in the conception and design of the study and acquisition of data and helped to draft the manuscript. PIJ participated in the conception of the study, acquisition and interpretation of data and helped to draft the manuscript. All authors read and approved the final manuscript.

## References

[B1] Cryer PE Physiology and pathophysiology of the human sympathoadrenal neuroendocrine system N Engl J Med 1980 17 436 444 10.1056/NEJM198008213030806 6248784

[B2] Dunser MW Hasibeder WR Sympathetic overstimulation during critical illness: adverse effects of adrenergic stress J Intensive Care Med 2009 17 293 316 10.1177/0885066609340519 19703817

[B3] Triposkiadis F Karayannis G Giamouzis G Skoularigis J Louridas G Butler J The sympathetic nervous system in heart failure physiology, pathophysiology, and clinical implications J Am Coll Cardiol 2009 17 1747 1762 10.1016/j.jacc.2009.05.015 19874988

[B4] Johansson PI Ostrowski SR Acute coagulopathy of trauma: balancing progressive catecholamine induced endothelial activation and damage by fluid phase anticoagulation Med Hypotheses 2010 17 564 567 10.1016/j.mehy.2010.07.031 20708846

[B5] Makhmudov RM Mamedov Y Dolgov VV Repin VS Catecholamine-mediated injury to endothelium in rabbit perfused aorta: a quantitative analysis by scanning electron microscopy Cor Vasa 1985 17 456 463 4092474

[B6] Kristova V Kriska M Canova R Hejdova E Kobzova D Dobrocky P Endothelial changes following repeated effect of vasoconstrictive substances in vitro Acta Physiol Hung 1993 17 363 370 8067251

[B7] Johansson PI Stensballe J Rasmussen LS Ostrowski SR High circulating adrenaline levels at admission predict increased mortality after trauma J Trauma Acute Care Surg 2012 17 428 436 22439205 10.1097/ta.0b013e31821e0f93

[B8] Ostrowski SR Sørensen AM Windeløv NA Perner A Welling KL Wanscher M Larsen CF Johansson PI High levels of soluble VEGF receptor 1 early after trauma are associated with shock, sympathoadrenal activation, glycocalyx degradation and inflammation Scand J Trauma Resusc Emerg Med 2012 17 27 10.1186/1757-7241-20-27 22490186 PMC3352319

[B9] Johansson PI Stensballe J Rasmussen LS Ostrowski SR A high admission syndecan-1 level, a marker of endothelial glycocalyx degradation, is associated with inflammation, protein C depletion, fibrinolysis, and increased mortality in trauma patients Ann Surg 2011 17 194 200 10.1097/SLA.0b013e318226113d 21772125

[B10] Rehm M Bruegger D Christ F Conzen P Thiel M Jacob M Chappell D Stoeckelhuber M Welsch U Reichart B Peter K Becker BF Shedding of the endothelial glycocalyx in patients undergoing major vascular surgery with global and regional ischemia Circulation 2007 17 1896 1906 10.1161/CIRCULATIONAHA.106.684852 17923576

[B11] Karlsberg RP Cryer PE Roberts R Serial plasma catecholamine response early in the course of clinical acute myocardial infarction: relationship to infarct extent and mortality Am Heart J 1981 17 24 29 10.1016/0002-8703(81)90408-7 7246410

[B12] Richardt G Munch G Neumann FJ Rauch B Kurz T Systemic and cardiac catecholamines during elective PTCA and during immediate PTCA for acute myocardial infarction Basic Res Cardiol 1997 17 52 60 9062652 10.1007/BF00803757

[B13] von Kanel R Dimsdale JE Effects of sympathetic activation by adrenergic infusions on hemostasis in vivo Eur J Haematol 2000 17 357 369 10.1034/j.1600-0609.2000.065006357.x 11168493

[B14] Hawkey CM Britton BJ Wood WG Peele M Irving MH Changes in blood catecholamine levels and blood coagulation and fibrinolytic activity in response to graded exercise in man Br J Haematol 1975 17 377 384 10.1111/j.1365-2141.1975.tb01835.x 1191556

[B15] Sefrin P Catecholamines in the serum of multiple trauma patients--mediators of ARDS? Prog Clin Biol Res 1987 17 477 486 3615447

[B16] van der PT Levi M Dentener M Jansen PM Coyle SM Braxton CC Buurman WA Hack CE ten Cate JW Lowry SF Epinephrine exerts anticoagulant effects during human endotoxemia J Exp Med 1997 17 1143 1148 10.1084/jem.185.6.1143 9091588 PMC2196238

[B17] Johansson PI Stissing T Bochsen L Ostrowski SR Thrombelastography and tromboelastometry in assessing coagulopathy in trauma Scand J Trauma Resusc Emerg Med 2009 17 45 10.1186/1757-7241-17-45 19775458 PMC2758824

[B18] Ostrowski SR Sorensen AM Larsen CF Johansson PI Thrombelastography and biomarker profiles in acute coagulopathy of trauma: A prospective study Scand J Trauma Resusc Emerg Med 2011 17 64 10.1186/1757-7241-19-64 22029598 PMC3212903

[B19] Schochl H Cadamuro J Seidl S Franz A Solomon C Schlimp CJ Ziegler B Hyperfibrinolysis is common in out-of-hospital cardiac arrest: Results from a prospective observational thromboelastometry study Resuscitation 2012 doi: 10.1016/j.resuscitation.2012.08.318 10.1016/j.resuscitation.2012.08.318 22922072

[B20] Gando S Nanzaki S Morimoto Y Kobayashi S Kemmotsu O Out-of-hospital cardiac arrest increases soluble vascular endothelial adhesion molecules and neutrophil elastase associated with endothelial injury Intensive Care Med 2000 17 38 44 10.1007/s001340050009 10663278

[B21] Grundmann S Fink K Rabadzhieva L Bourgeois N Schwab T Moser M Bode C Busch HJ Perturbation of the endothelial glycocalyx in post cardiac arrest syndrome Resuscitation 2012 17 715 720 10.1016/j.resuscitation.2012.01.028 22306259

[B22] Becker BF Chappell D Bruegger D Annecke T Jacob M Therapeutic strategies targeting the endothelial glycocalyx: acute deficits, but great potential Cardiovasc Res 2010 17 300 310 10.1093/cvr/cvq137 20462866

[B23] Salmon AH Satchell SC Endothelial glycocalyx dysfunction in disease: albuminuria and altered microvascular permeability J Pathol 2012 17 562 574 10.1002/path.3964 22102407

[B24] Aird WC The role of the endothelium in severe sepsis and multiple organ dysfunction syndrome Blood 2003 17 3765 3777 10.1182/blood-2002-06-1887 12543869

[B25] Haywood-Watson R Pati S Kozar R Faz J Holcomb JB Gonzalez E Human micro-vascular barrier disruption after hemorrhagic shock J Surg Res 2010 17 313

[B26] Kozar RA Peng Z Zhang R Holcomb JB Pati S Park P Ko TC Paredes A Plasma restoration of endothelial glycocalyx in a rodent model of hemorrhagic shock Anesth Analg 2011 17 1289 1295 10.1213/ANE.0b013e318210385c 21346161 PMC3102787

[B27] Haywood-Watson RJ Holcomb JB Gonzalez EA Peng Z Pati S Park PW Wang W Zaske AM Menge T Kozar RA Modulation of syndecan-1 shedding after hemorrhagic shock and resuscitation PLoS ONE 2011 17 e23530 10.1371/journal.pone.0023530 21886795 PMC3158765

[B28] Ishii H Uchiyama H Kazama M Soluble thrombomodulin antigen in conditioned medium is increased by damage of endothelial cells Thromb Haemost 1991 17 618 623 1651569

[B29] Blann A Seigneur M Soluble markers of endothelial cell function Clin Hemorheol Microcirc 1997 17 3 11 9181753

[B30] Boffa MC Considering cellular thrombomodulin distribution and its modulating factors can facilitate the use of plasma thrombomodulin as a reliable endothelial marker? Haemostasis 1996 17 Suppl 4 233 243 8979129 10.1159/000217304

[B31] Lindberg S Pedersen SH Mogelvang R Bjerre M Frystyk J Flyvbjerg A Galatius S Jensen JS Usefulness of adiponectin as a predictor of all cause mortality in patients with ST-segment elevation myocardial infarction treated with primary percutaneous coronary intervention Am J Cardiol 2012 17 492 496 10.1016/j.amjcard.2011.09.041 22105783

[B32] Aird WC Endothelium as an organ system Crit Care Med 2004 17 S271 S279 10.1097/01.CCM.0000129669.21649.40 15118530

[B33] Nieuwdorp M van Haeften TW Gouverneur MC Mooij HL van Lieshout MH Levi M Meijers JC Holleman F Hoekstra JB Vink H Kastelein JJ Stroes ES Loss of endothelial glycocalyx during acute hyperglycemia coincides with endothelial dysfunction and coagulation activation in vivo Diabetes 2006 17 480 486 10.2337/diabetes.55.02.06.db05-1103 16443784

[B34] Salomaa V Matei C Aleksic N Sansores-Garcia L Folsom AR Juneja H Chambless LE Wu KK Soluble thrombomodulin as a predictor of incident coronary heart disease and symptomless carotid artery atherosclerosis in the Atherosclerosis Risk in Communities (ARIC) Study: a case-cohort study Lancet 1999 17 1729 1734 10.1016/S0140-6736(98)09057-6 10347984

[B35] Blann AD McCollum CN Lip GY Relationship between plasma markers of endothelial cell integrity and the Framingham cardiovascular disease risk-factor scores in apparently healthy individuals Blood Coagul Fibrinolysis 2002 17 513 518 10.1097/00001721-200209000-00006 12192303

[B36] Seigneur M Dufourcq P Conri C Constans J Mercie P Pruvost A Amiral J Midy D Baste JC Boisseau MR Levels of plasma thrombomodulin are increased in atheromatous arterial disease Thromb Res 1993 17 423 431 10.1016/0049-3848(93)90116-6 8134903

[B37] Omland T Aarsland T Aakvaag A Lie RT Dickstein K Prognostic value of plasma atrial natriuretic factor, norepinephrine and epinephrine in acute myocardial infarction Am J Cardiol 1993 17 255 259 10.1016/0002-9149(93)90669-4 8342501

[B38] Katayama T Nakashima H Furudono S Honda Y Suzuki S Yano K Evaluation of neurohumoral activation (adrenomedullin, BNP, catecholamines, etc.) in patients with acute myocardial infarction Intern Med 2004 17 1015 1022 10.2169/internalmedicine.43.1015 15609694

[B39] Dutta P Courties G Wei Y Leuschner F Gorbatov R Robbins CS Iwamoto Y Thompson B Carlson AL Heidt T Majmudar MD Lasitschka F Etzrodt M Waterman P Waring MT Chicoine AT van der Laan AM Niessen HWM Piek JJ Rubin BB Butany J Stone JR Katus HA Murphy SA Morrow DA Sabatine MS Vinegoni C Moskowitz MA Pittet MJ Libby P Myocardial infarction accelerates atherosclerosis Nature 2012 17 325 329 10.1038/nature11260 22763456 PMC3401326

[B40] Oswald GA Smith CC Betteridge DJ Yudkin JS Determinants and importance of stress hyperglycaemia in non-diabetic patients with myocardial infarction Br Med J (Clin Res Ed) 1986 17 917 922 10.1136/bmj.293.6552.917 PMC1341710 3094714

[B41] Leineweber K Heusch G Beta 1-and beta 2-adrenoceptor polymorphisms and cardiovascular diseases Br J Pharmacol 2009 17 61 69 10.1111/j.1476-5381.2009.00187.x 19422376 PMC2795234

[B42] Benedict CR Shelton B Johnstone DE Francis G Greenberg B Konstam M Probstfield JL Yusuf S Prognostic significance of plasma norepinephrine in patients with asymptomatic left ventricular dysfunction. SOLVD Investigators Circulation 1996 17 690 697 10.1161/01.CIR.94.4.690 8772689

[B43] Hartmann F Kurowski V Maghsoudi A Kurz T Schwarz M Bonnemeier H Tolg R Jain D Wiegand U Katus H Richardt G Plasma catecholamines and N-terminal proBNP in patients with acute myocardial infarction undergoing primary angioplasty. Relation to left ventricular function and clinical outcome Z Kardiol 2003 17 73 81 10.1007/s00392-003-0885-8 12545304

